# Reactive Case Detection for *Plasmodium vivax* Malaria Elimination in Rural Amazonia

**DOI:** 10.1371/journal.pntd.0005221

**Published:** 2016-12-12

**Authors:** Pablo S. Fontoura, Bruna F. Finco, Nathália F. Lima, Jaques F. de Carvalho, Joseph M. Vinetz, Márcia C. Castro, Marcelo U. Ferreira

**Affiliations:** 1 Department of Parasitology, Institute of Biomedical Sciences, University of São Paulo, São Paulo, SP, Brazil; 2 Division of Infectious Diseases, Department of Medicine, University of California San Diego, La Jolla, CA, United States of America; 3 Alexander von Humboldt Institute of Tropical Medicine and Faculty of Sciences, Department of Cellular and Molecular Sciences, Laboratory of Research and Development, Universidad Peruana Cayetano Heredia, Lima, Peru; 4 Department of Global Health and Population, Harvard T. H. Chan School of Public Health, Boston, MA, United States of America; Johns Hopkins Bloomberg School of Public Health, UNITED STATES

## Abstract

**Background:**

Malaria burden in Brazil has reached its lowest levels in 35 years and *Plasmodium vivax* now accounts for 84% of cases countrywide. Targeting residual malaria transmission entrenched in the Amazon is the next major challenge for ongoing elimination efforts. Better strategies are urgently needed to address the vast reservoir of asymptomatic *P*. *vivax* carriers in this and other areas approaching malaria elimination.

**Methods:**

We evaluated a reactive case detection (RCD) strategy tailored for *P*. *vivax* transmission in farming settlements in the Amazon Basin of Brazil. Over six months, 41 cases detected by passive surveillance triggered four rounds of RCD (0, 30, 60, and 180 days after index case enrollment), using microscopy- and quantitative real-time polymerase chain reaction (qPCR)-based diagnosis, comprising subjects sharing the household (HH) with the index case (n = 163), those living in the 5 nearest HHs within 3 km (n = 878), and individuals from 5 randomly chosen control HHs located > 5 km away from index cases (n = 841). Correlates of infection were identified with mixed-effects logistic regression models. Molecular genotyping was used to infer local parasite transmission networks.

**Principal findings/Conclusions:**

Subjects in index and neighbor HHs were significantly more likely to be parasitemic than control HH members, after adjusting for potential confounders, and together harbored > 90% of the *P*. *vivax* biomass in study subjects. Clustering patterns were temporally stable. Four rounds of microscopy-based RCD would identify only 49.5% of the infections diagnosed by qPCR, but 76.8% of the total parasite biomass circulating in the proximity of index HHs. However, control HHs accounted for 27.6% of qPCR-positive samples, 92.6% of them from asymptomatic carriers beyond the reach of RCD. Molecular genotyping revealed high *P*. *vivax* diversity, consistent with complex transmission networks and multiple sources of infection within clusters, potentially complicating malaria elimination efforts.

## Author Summary

Addressing the vast reservoir of asymptomatic *Plasmodium vivax* carriers clustered in hard-to-reach rural communities is a major challenge faced by countries approaching malaria elimination across Latin America and Asia. Routine surveillance targets subjects presenting with fever or reporting recent fever, but overlooks asymptomatic infections that might be otherwise detected by periodic mass blood surveys of the entire population at risk. Here we show that subjects living in close proximity to malaria cases detected by routine passive surveillance are much more likely to carry both symptomatic and asymptomatic infections than randomly selected inhabitants in the same farming settlements in the Amazon Basin of Brazil. Four rounds of microscopy-based screening for malaria parasites targeted at these high-risk subjects would identify 49.5% of the parasite carriers (who together harbored 76.8% of the total *P*. *vivax* biomass circulating in the proximity of index cases) detected by a more sensitive molecular method. Whether subpatent and asymptomatic carriers outside the identified clusters of symptomatic infections, beyond the reach of our screening, represent a significant parasite reservoir remains undetermined. The extensive genetic diversity found in local *P*. *vivax* populations suggests that multiple sources of infection fuel ongoing residual transmission within malaria clusters, further complicating current elimination efforts.

## Introduction

Over one-third of the world's population is currently at risk of infection with *Plasmodium vivax*, the human malaria parasite with the widest global distribution [1]. Where both species coexist, *P*. *vivax* typically causes less severe cases and deaths than *P*. *falciparum*, but its distinctive biological features pose major challenges for malaria elimination strategies focused on early diagnosis and prompt treatment of blood-stage infections. First, low-density *P*. *vivax* infections, which are often subpatent (i.e., missed by conventional microscopy) and asymptomatic, predominate in areas approaching malaria elimination, rendering accurate clinical and laboratory diagnosis more difficult [2]. Accordingly, 54% of the *P*. *vivax* infections detected by polymerase chain reaction (PCR) in a rural Amazonian cohort were missed by microscopy; 57% of them caused no clinical signs or symptoms suggestive of malaria and 33% were both subpatent and asymptomatic [3]. Second, *P*. *vivax* remains silent within human hosts for several months as hypnozoites, the dormant liver stages that may eventually cause relapses [4]. Finally, compared to other human malaria parasites, *P*. *vivax* transmission is favored by the earlier production within human hosts of blood stages that are infective to mosquito vectors (mature gametocytes) as well as by the faster development within vectors of stages that are infective to humans (sporozoites) [5].

Brazil is one of the 14 (out of 21) malaria-endemic countries in the region of the Americas that achieved a ≥ 75% reduction in their case incidence rates between 2000 and 2015, as called for by the United Nations' Millennium Development Goals [6]. This country currently contributes 37% of the regional malaria burden, with 142,314 microscopically confirmed cases in 2015 (http://portalsaude.saude.gov.br/index.php/o-ministerio/principal/leia-mais-o-ministerio/662-secretaria-svs/vigilancia-de-a-a-z/malaria/11346-situacao-epidemiologica-dados). Most transmission hotspots are located in remote sites across the Amazon Basin. This vast region, with 60% of the country's territory but only 13% of its total population, accounts for 99.5% of all malaria cases diagnosed in Brazil; 84% of them are due to *P*. *vivax* [7].

Eliminating residual foci when malaria is steadily declining and reaching pre-elimination stages is the next major challenge in Brazil [7]. Routine surveillance targets subjects presenting with fever or with a history of recent fever or other malaria-related symptoms and signs, who are screened for malaria parasites by microscopy or rapid diagnostic tests (RDT) followed by antimalarial treatment if found to be infected [8]. Accordingly, nearly 60% of all infections in Brazil are laboratory-confirmed within 48 hours after the onset of symptoms, preventing severe morbidity [7]. However, this strategy overlooks asymptomatic infections that might be otherwise detected by periodic cross-sectional surveys of the entire population at risk [9]. As malaria transmission declines, large populations must be screened to diagnose relatively few asymptomatic carriers, and diagnostic techniques available for large-scale use, such as microscopy and RDT, are not sensitive enough to detect low-grade infections that are typical of residual malaria settings [10].

Here, we evaluate a reactive case detection (RCD) strategy [11] that has been tailored for rural Amazonian communities with low *P*. *vivax* endemicity and substantial spatial clustering of malaria episodes [12,13]. We sought to test three hypotheses: (a) individuals living in close proximity to malaria cases diagnosed by routine surveillance (index cases) are more likely to carry malaria parasites (including subpatent and asymptomatic infections) than randomly selected inhabitants living in the same area; (b) the increased risk of infection in the proximity of index cases persists over the next few months, due to the relapsing nature of *P*. *vivax*; and (c) molecular genotyping of parasites may help to determine the epidemiological connections among cases found by RCD.

## Methods

### Study area

Acrelândia is a municipality situated in the easternmost corner of Acre State, Amazon Basin of Brazil, bordering with the States of Amazonas and Rondônia (S1 Fig). The municipality covers a territory of 1,808 km^2^ between the Abunã and Iquiri Rivers, in the Acre River Valley, with a population of 14,120 inhabitants. Nearly half of them live in the town of Acrelândia (9°49'40''S, 66°52'58''W), located 35 km northeast of Plácido de Castro (the nearest urban center, on the border with Bolivia) and 112 km east of Rio Branco (capital city of Acre). With its equatorial humid climate, this area receives the most rainfall (annual average, 2,198.5 mm) between December and March. The mean annual temperature is 24.5°C. Subsistence agriculture and cattle raising are the main economic activities, with coffee, bananas, and rice being the predominant cash crops.

Acrelândia has experienced a major decline in malaria incidence in recent years [14], but year-round residual malaria transmission persists in farming settlements surrounding the town, with 163 slide-confirmed cases in 2009, 248 in 2010, 104 in 2011 and 79 in 2012 (http://portalsaude.saude.gov.br/index.php/o-ministerio/principal/leia-mais-o-ministerio/662-secretaria-svs/vigilancia-de-a-a-z/malaria/11346-situacao-epidemiologica-dados). Although both *P*. *falciparum* and *P*. *vivax* were transmitted in the early 2000s, with the latter accounting for over 80% of all infections [14], only *P*. *vivax* has been observed in slide-confirmed indigenous cases since 2010. Selective indoor spraying with residual insecticides, implemented in early 2008, and the widespread distribution of long-lasting insecticide-treated bed nets, since 2010, are likely to have contributed to the dramatic reduction of malaria transmission (with the virtual elimination of *P*. *falciparum*) in Acrelândia in recent years. The main local vector is the highly anthropophilic and exophilic *Anopheles darlingi* [15], but *An*. *benarrochi* s.l., *An*. *oswaldoi* s.l., and *An*. *konderi* s.l. are also often found in larval habitats [16].

### Study design and population

**Fig 1:**
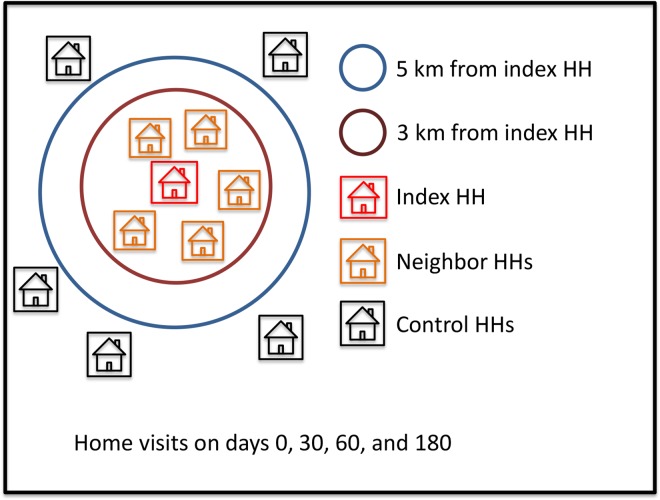
Schematic representation of the study design. For each index case detected by routine passive surveillance and enrolled in the study we invited the following individuals to participate: (a) all subjects who shared the household (HH) with the index case (index HH, represented in red), (b) all members of the five nearest neighbors within a radius of 3 km from the index HH (neighbor HHs, represented in orange), and (c) randomly chosen subjects living in five control HHs > 5 km away from the index HH but still in the same or nearby locality (control HHs, represented in black). Four visits were made to each HH, the first at the time of index case diagnosis (day 0) and the following 30, 60, and 180 days later.

The study design is summarized in Fig 1. Briefly, the RCD strategy was triggered whenever an eligible malaria case was detected by routine passive surveillance (“index case”); subjects who shared the household (HH) with the index case (“index HH” members) and their neighbors within a defined radius (“neighbor HH” members) were screened for malaria parasites in search of secondary infections. We also enrolled randomly chosen subjects in “control HHs” located beyond a 5-km radius from index HHs, but in the same or nearby locality, to serve as controls putatively unexposed to secondary infections originating from the vicinity of index cases.

The vast majority of the 1,923 study participants were enrolled between January and July 2013, except for those who were later recruited to replace individuals leaving the site before study completion (see below). Inclusion criteria were: (a) all subjects aged ≥ 3 months living in the selected HHs who provided written informed consent, or with parental written informed consent and assent when age-appropriate; and (b) subjects with plans to remain in the study site for the next six months. Exclusion criteria included mental disorders and any chronic or acute condition that, in the opinion of the field nurse, might affect the results of the study or the subjects' ability of understanding the study objectives and of providing informed consent. Participants were classified into the following categories: (a) 41 index cases—had a *P*. *vivax* infection diagnosed by routine surveillance during the enrollment period and were recruited in one of the two only government-run malaria clinics currently operating in Acrelândia (one in the urban area and one on the BR 364 highway, next to the border with the State of Rondônia; no other public or private facilities provide malaria diagnosis and treatment in this area); (b) 163 index HH members—subjects who shared the HH with the index case; (c) 878 neighbor HH members—subjects living in the five nearest houses, within a radius of up to 3 km (average, 731 m) away from the index HH (neighbor HH members); (d) 841 control HH members—subjects living in five randomly chosen houses located in the same or nearby locality but > 5 km (average, 12 km) away from the index HH. The 5-km radius was chosen based on the maximal flight range reported for *An*. *darlingi* in the Amazon [17] to ensure that control subjects are unlikely to share common sources of infection with index cases and their neighbors.

Home visits were made to eligible subjects on day 0 (at enrollment, within 48 hrs of the index case diagnosis) and 30 (±5), 60 (±5), and 180 (±10) days after the first visit, with a total of 6,172 observations. Visits were scheduled to maximize the probability of finding infections that had been acquired at the same time as the index case (day 0), those that were secondary to the index case or other infections diagnosed at baseline (day 30), new infections originating from these secondary infections (day 60), as well as most relapses, which are usually recorded in local strains of *P*. *vivax* up to 180 days after the primary infection [18]. In every visit, subjects were interviewed by our field team, consisting of two biologists and a nurse, and invited to provide a 5-mL venous blood sample (index cases) or finger-prick blood sample (all other study participants) for malaria diagnosis by both thick-smear microscopy and quantitative real-time PCR (qPCR), regardless of any clinical symptoms (S1 Fig). During home visits, 6,028 blood samples were collected, with only 144 (2.4%) refusals. Neighbor or control HH members who left the study site over the six-month study period (n = 151) were replaced with their nearest neighbors available.

### Laboratory methods

Laboratory diagnosis of malaria was based on microscopic examination of thick smears and qPCR. A total of 6,028 thick blood smears were stained with Giemsa and examined for malaria parasites under 1000 × magnification in our laboratory in the town of Acrelândia. At least 100 fields (corresponding to approximately 0.3 μL of blood) were examined before a slide was declared negative. We additionally used qPCR to detect and quantify *P*. *vivax* parasitemia on 5,969 clinical samples. To this end, we isolated DNA templates using Qiagen (Hilden, Germany) kits. We used QIAamp DNA blood kits for 200-μL venous blood samples and QIAmp DNA micro kits for 5-μL capillary blood samples eluted from three 3-mm bloodspots excised from FTA Micro Cards (Whatman, Clifton, NJ). Final DNA elution volumes were 20 μL for bloodspots and 200 μL for whole blood. Each 20 μL qPCR mixture contained 2 μL of template DNA (corresponding to approximately 2 μL of venous blood or 0.5 μL of finger-prick blood), 10 μL of 2× Maxima SYBR Green qPCR master mixture (Fermentas, Burlington, Canada) and 0.5 μM of each of the primers P1 (ACG ATC AGA TAC CGT CGT AAT CTT) and V1 (CAA TCT AAG AAT AAA CTC CGA AGA GAA A) [19], which allow the amplification of a 100-base pair (bp) fragment of the *P*. *vivax* 18S rRNA gene. Standard curves were prepared with serial tenfold dilutions of the target sequence, cloned into pGEM-T Easy vectors (Promega, Madison, WI), to allow for parasite load quantitation (number of parasites/μL of blood). We used a Step One Plus Real-Time PCR System (Applied Biosystems, Foster City, CA) for PCR amplification with a template denaturation step at 95°C for 10 min, followed by 40 cycles of 15 sec at 95°C and 1 minute at 60°C, with fluorescence acquisition at the end of each extension step. Amplification was followed by a melting program consisting of 15 sec at 95°C, 15 sec at 60°C, and a stepwise temperature increase of 0.2°C/sec until 95°C, with fluorescence acquisition at each temperature transition. The detection threshold of this diagnostic qPCR is approximately 3.3 parasites/μL of blood. No-template controls (containing all reagents for amplification except for the DNA template) were run for every qPCR microplate. To estimate the relative contribution of particular subgroups of infected individuals to the total *P*. *vivax* biomass found in the study population, we summed up all individual qPCR-derived *P*. *vivax* densities and calculated the fraction corresponding to each subgroup. Because parasite densities are expressed as parasites per blood volume, we assumed that average whole-body blood volumes (volemias) do not differ across subgroups.

**Fig 2:**
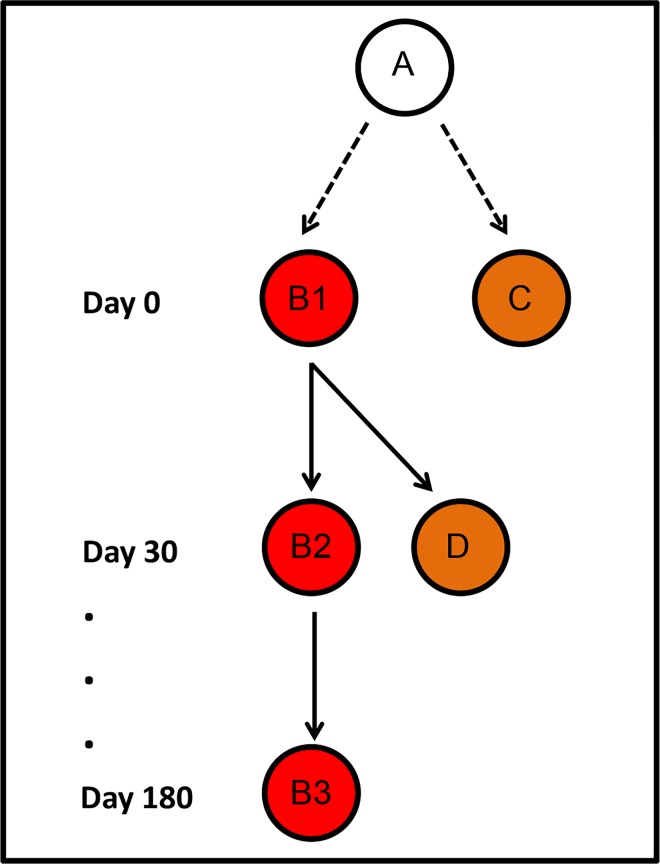
Inferring transmission pathways of *Plasmodium vivax* genotypes within malaria clusters. Circles represent individual study subjects carrying identical parasite genotypes; red and orange circles represent index and neighbor household (HH) members, respectively. Infections B1 and C, both diagnosed on day 0, have a common source (A) that had not been included in the study. D is a secondary infection with known source within the cluster (namely, B1). B2 and B3 have been diagnosed in the same subject as B1, 30 and 180 days later. If B1 was treated with chloroquine-primaquine (as all microscopy-positive infections were in our study), B2 may be either a recrudescence (due to chloroquine failure) or an early homologous relapse (due to primaquine failure to clear liver-stage hypnozoites); if B1 was missed by onsite microscopy and left untreated during our study, B2 may also represent a chronic, untreated blood-stage infection. B3 may represent either a chronic untreated infection (if B1 and B2 were left untreated) or a late homologous relapse (if chloroquine has successfully cleared blood-stage parasites but primaquine failed to clear hypnozoites).

Parasite genotyping was carried out on microscopy- and qPCR-positive samples to classify infections as either (a) recrudescences, homologous relapses or persisting untreated infections (identical multilocus genotype reappearing in repeated infections in the same subject), (b) related infections in different subjects (identical or similar genotypes appearing in different subjects either in the same or consecutive RCD rounds, suggestive of local—within cluster—transmission) or (c) unrelated, sporadic infections or heterologous relapses (different genotypes), as described in Fig 2. Note that relapses may originate from reactivation of either the same parasite clone found in the primary bloodstream infection (homologous hypnozoites) or another, genetically different clone (heterologous hypnozoites) [20,21]. We used nested PCR [22] to genotype three highly polymorphic single-copy markers: one variable domain of the *merozoite surface protein-1* gene (*msp1*F3 [23]) and two microsatellite DNA markers, *Pv3*.*27* [24] and *MS16* [25]. PCR products were analyzed by capillary electrophoresis on an automated DNA sequencer ABI 3500 (Applied Biosystems), and their lengths (in bp) and relative abundance (peak heights in electropherograms) were determined using the commercially available GeneMapper 4.1 (Applied Biosystems) software. DNA samples from 361 laboratory-confirmed *P*. *vivax* infections diagnosed during the study (66 from index cases and 295 from other study participants) were tested; 343 (95.0%) of them were successfully genotyped at all 3 loci. The minimal detectable peak height was set to 200 arbitrary fluorescence units. Because stutter bands may be occasionally observed in microsatellite genotyping, we scored two alleles at a locus only when the minor peak was > 33% the height of the predominant peak. Only two samples, out of 343 that were fully genotyped, had minor alleles with peaks of > 200 fluorescence units that were discarded (one in each sample) because they failed to reach one-third of the height of the major peak in the same sample. Samples were considered to contain multiple clones if at least one locus showed more than one allele. Multilocus genotypes were defined as unique combinations of alleles at each locus analyzed. Although our descriptive analysis considered the most abundant allele (as judged by peak heights) at each locus for assigning predominant genotypes to multiple-clone infections, we also considered all possible genotypes in pairwise comparisons of samples from the same subjects (recurring infections) or within the same cluster (comprising the index and neighbor HHs). For this purpose, we listed all combinations of alleles, considering major and minor peaks, in each pair. Samples were considered to harbor *identical* parasites when they had exactly the same predominant (or only) genotype; they were considered to harbor *similar* parasites when the same alleles (considering major and minor peaks) were found at all loci, but their predominant genotypes were different.

### Clinical definitions and treatment

A laboratory-confirmed *P*. *vivax* infection was defined as any episode of parasitemia detected by thick-smear microscopy, qPCR, or both. Subpatent infections were defined as infections confirmed by qPCR but missed by microscopy. We defined symptomatic malaria as a laboratory-confirmed infection, irrespective of the parasite density, diagnosed in a subject reporting one or more signs and symptoms investigated (fever, headache, arthralgia, myalgia, and lower back pain), up to seven days before the interview. Subjects with laboratory-confirmed infections, irrespective of the parasite density, but no malaria-related signs or symptoms up to seven days prior to the interview, were classified as asymptomatic parasite carriers. All study participants with vivax malaria infection confirmed by onsite microscopy, either symptomatic or not, received free-of-charge treatment with chloroquine (total dose, 25 mg of base/kg over 3 days) and primaquine (0.5 mg of base/kg/day for 7 days) following the latest antimalarial therapy guidelines of the Ministry of Health of Brazil [26]. According to these guidelines, infections in microscopy-negative subjects that were later detected by qPCR were not routinely treated.

### Statistical analysis

Data were entered and cleaned using EpiInfo version 3.5.2 software (Center for Diseases Control and Prevention, Atlanta, GA) and analyzed with STATA version 14.1 software (Stata, College Station, TX). Proportions were compared by applying standard χ^2^ of Fisher exact tests tests to contingency tables. Statistical significance was defined at the 5% level (two-tailed tests) and 95% confidence intervals (CI) were estimated whenever appropriate.

Regression models were run to identify correlates of *P*. *vivax* infection in one or more RCD rounds, regardless of any symptoms, stratified by diagnostic method: microscopy (n = 5,866 complete observations) and qPCR (n = 5,807 complete observations) in one or more RCD rounds, regardless of any symptoms. Index cases were excluded from this analysis. Individual-level variables included in the model were: age, gender, occupation, past migratory history, travel history during the last month, overnight stays in the forest in the last month (for hunting or fishing), use of vector control measures such as bed nets, history of slide-confirmed *P*. *vivax* malaria within the past six months, history of malaria-related symptoms and signs within the previous week, and use of antimalarials within the past month. HH-level variables were: HH location, determined with the use of global positioning system (GPS) receivers, and HH size. S1 Database, provided as supplementary material online, has the variables included in the logistic regression models.

Due to the nested structure of the data, which introduces dependency among observations that can affect model parameter estimates, we used the “melogit” STATA command to build mixed-effects logistic regression models that included the grouping variables as random factors. We had repeated observations (on days 0, 30, 60, and 180; grouping variable, “round”) nested within subjects (grouping variable, “individual”) who were clustered within HHs (grouping variable, “household”). Finally, HHs were clustered within index cases (grouping variable, “index case”), since the inclusion of each index case triggered the recruitment of matched study participants (other members of the index HHs, neighbor HHs, and control HHs; Fig 1). Covariates were introduced in the models in a stepwise forward approach, and only those that were associated with the outcome at a significance level of at least 20% were retained in the final model. Cases with missing information were excluded. In addition, we fitted mixed-effects Poisson regression models to the data, but the random-effects variances associated with the estimates were substantially higher than those obtained with the logistic models described above. Thus, here we only present results derived from the logistic regression analysis.

As required for all observational studies published by *PLoS Neglected Tropical Diseases*, this article includes the STROBE (**STrengthening the Reporting of OBservational studies in Epidemiology)** checklist to document its compliance with STROBE guidelines (S1 Checklist).

### Ethics statement

Study protocols were approved by the Institutional Review Board of the Institute of Biomedical Sciences, University of São Paulo (approval number, 228.720). Written informed consent was obtained from all study participants or their parents/guardians.

## Results

### Subject characteristics

**Fig 3:**
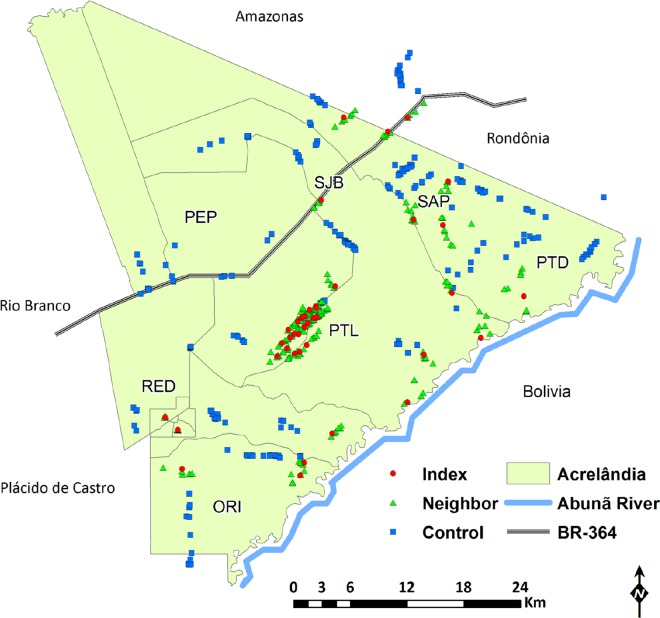
Spatial distribution of individual index, neighbor, and control households (HHs) included in the study. HHs were distributed into seven localities of the municipality of Acrelândia (defined by the local malaria control program): Gleba Porto Luiz (PTL), Projeto Santo Antônio Peixoto (SAP), São João do Balanceio (SJB), Projeto Redenção (RED), Projeto Orion (ORI), Reserva Porto Dias (PTD), and Projeto Pedro Peixoto (PEP). Note that one index HH, five neighbor HHs, and 29 control HHs are situated outside the territory of Acrelândia (light green area), but still within the catchment area of local health services. The map also shows the nearest municipalities, Plácido de Castro and Rio Branco, the Abunã River, which delimits the border with Bolivia, and the BR 364 interstate highway, which connects the States of Acre, Rondônia, and southern Amazonas to the rest of the country.

The study population comprised 1,923 subjects (13.6% of the total population of Acrelândia), who were aged between 3 months and 89 years (mean, 26.5 years) and had been living in the study site for 1 month to 64 years (mean, 9 years). Most (1,037 or 53.9%) were males and 1,613 (83.9%) were born in the Amazon. Nearly half of them reported previous slide-confirmed malaria episodes, but <5% had a *P*. *vivax* infection within the past six months. Eighteen percent reported overnight stays in the forest in the last month. Study participants were distributed into seven localities of the municipality of Acrelândia: Gleba Porto Luiz (PTL), Projeto Santo Antônio Peixoto (SAP), Projeto São João do Balanceio (SJB), Projeto Redenção (RED), Projeto Orion (ORI), Reserva Porto Dias (PTD), and Projeto Pedro Peixoto (PEP). Individual HH locations are shown in Fig 3. The 41 index cases corresponded to 21.9% of all *P*. *vivax* infections diagnosed in Acrelândia between January and July 2013; between 19 (March) and 98 (July) cases were diagnosed monthly during the study period. Index cases were tentatively classified as indigenous, relapsing, or imported according to classical eradication-era criteria [27]; 6 were considered imported (cases that could be traced to a locality outside the study site), 11 were putative relapses (cases diagnosed in subjects with a history of *P*. *vivax* infection within the past six months), and 24 were considered indigenous (cases that were locally acquired or autochthonous). Index cases, other index HH members and neighbor HH members were recruited in all localities listed above, except for PEP; 22 (53.6%) index cases lived in PTL.

### Microscopy positivity rates in RCD rounds

A total of 108 infections were diagnosed by microscopy by our field team over the entire study period. *Plasmodium vivax* positivity rates differed significantly across HH types (Table 1). They were relatively high (range, 4.1–8.2%) in index HHs (35 positive slides, 6.1%, among 577 examined, 12 of them from asymptomatic subjects; index cases excluded) and much lower (0.0–0.3%) in control HHs (3 positive slides, 0.1%, among 2,586 examined, one of them from an asymptomatic carrier). No slide was positive for *P*. *falciparum* or *P*. *malariae*. These results indicate that index HH members are much more likely to carry patent *P*. *vivax* parasitemias than randomly selected inhabitants living in the same area (control HH members); interestingly, the highest prevalence of infection was observed in index HHs not only at the first visit but also over the following RCD rounds.

**Table 1:** Absolute and relative frequency of symptomatic and asymptomatic *Plasmodium vivax* infections diagnosed by conventional microscopy among members of index, neighbor, and control households (HHs) in four rounds of reactive case detection (RCD) in Acrelândia, Brazil, 2013.

RCD round	Type of infection	Index HHs		Neighbor HHs		Control HHs		*P-*value^a^
		Number of infections	%	Number of infections	%	Number of infections	%	
1 (Day 0)	Symptomatic	7	4.8%	5	0.7%	0	0.0%	< 0.001
	Asymptomatic	5	3.4%	0	0.0%	1	0.2%	< 0.001
	Total^b^	12	8.2%	5	0.7%	1	0.2%	< 0.001
	No. tested	147		688		634		
2 (Day 30)	Symptomatic	7	4.9%	8	1.2%	0	0.0%	< 0.001
	Asymptomatic	0	0.0%	5	0.8%	0	0.0%	0.05
	Total^c^	7	4.9%	13	2.0%	0	0.0%	< 0.001
	No. tested	143		647		627		
3 (Day 60)	Symptomatic	6	4.3%	15	2.2%	0	0.0%	< 0.001
	Asymptomatic	4	2.8%	15	2.2%	0	0.0%	< 0.001
	Total^c^	10	7.1%	30	4.4%	0	0.0%	< 0.001
	No. tested	141		679		647		
4 (Day 180)	Symptomatic	3	2.0%	10	1.4%	2	0.3%	0.038
	Asymptomatic	3	2.0%	12	1.7%	0	0.0%	0.002
	Total^c^	6	4.1%	22	3.2%	2	0.3%	< 0.001
	No. tested	146		689		678		

^a^Two-tailed *P-*values for χ^2^ tests on 3 × 2 contingency tables testing the null hypothesis of similar proportions of positive results across rows.

^b^Pairwise comparisons of positivity rates across row with Fisher exact test (at 5% significance): index HHs > neighbor and control HHs; neighbor HHs = control HHs.

^c^Pairwise comparisons of positivity rates across row with Fisher exact test (at 5% significance): index and neighbor HHs > control HHs; index HHs = neighbor HHs.

Whether RCD strategies should target only index HHs or be extended to neighbor HHs remains a matter of debate. We found widely variable slide positivity rates in neighbor HHs across RCD rounds in our population, ranging from 0.7% (day 0) to 4.4% (day 60) (Table 1). In the first RCD round, slide-confirmed infections were as rare in neighbor HHs as they were in control HHs (Fisher exact test, *P* = 0.220), suggesting that targeting neighbor HH members on day 0 would not be more effective for diagnosing additional patent infections than randomly screening subjects living in the same locality. In contrast, positivity rates in all other RCD rounds were similar between index HHs and neighbor HHs (Fisher exact test, *P* > 0.05 in all comparisons) and significantly higher than those in control HHs (Fisher exact test, *P* < 0.001 in all comparisons), indicating that screening index and neighbor HH members would be similarly effective for diagnosing new infections between days 30 and 180 (Table 1). Overall, 70 slides (2.6%) from neighbor HHs were positive among 2,703 examined. Thirty-two (45.7%) positive slides were from asymptomatic carriers who were likely to be missed by routine malaria surveillance.

### qPCR positivity rates in RCD rounds

We next compared *P*. *vivax* prevalence diagnosed by qPCR in index HHs (excluding the index case), neighbor HHs and control HHs during the four RCD rounds (Table 2). Not surprisingly, qPCR detected more than double the number of infections than microscopy (293 positive samples among 5,807 examined). Overall qPCR positivity rates were 10.6% in index HHs (1.7-fold higher than microscopy), 5.7% in neighbor HHs (2.2-fold higher than microscopy), and 3.2% in control HHs (> 30-fold higher than microscopy). The vast majority (75 or 92.6%) of infections diagnosed by qPCR in control HHs were asymptomatic, consistent with a silent circulation of *P*. *vivax* in the community that would be entirely missed by RCD.

**Table 2:** Absolute and relative frequency of symptomatic and asymptomatic *Plasmodium vivax* infections diagnosed by quantitative real-time polymerase chain reaction (qPCR) among members of index, neighbor, and control households (HHs) in four rounds of reactive case detection (RCD) in Acrelândia, Brazil, 2013.

RCD round	Type of infection	Index HHs		Neighbor HHs		Control HHs		*P-*value^a^
		Number of infections	%	Number of infections	%	Number of infections	%	
1 (Day 0)	Symptomatic	6	4.1%	6	0.9%	2	0.3%	0.001
	Asymptomatic	16	10.9%	31	4.6%	33	5.2%	0.009
	Total^b^	22	15.0%	37	5.5%	35	5.5%	<0.001
	No. tested	147		674		631		
2 (Day 30)	Symptomatic	8	5.6%	9	1.4%	2	0.3%	<0.001
	Asymptomatic	10	7.0%	36	5.6%	33	5.3%	0.721
	Total^b^	18	12.6%	45	7.0%	35	5.6%	0.012
	No. tested	143		645		626		
3 (Day 60)	Symptomatic	4	2.9%	16	2.4%	0	0.0%	<0.001
	Asymptomatic	7	5.0%	19	2.9%	2	0.3%	<0.001
	Total^c^	11	7.9%	35	5.3%	2	0.3%	<0.001
	No. tested	139		660		634		
4 (Day 180)	Symptomatic	4	2.7%	13	1.9%	2	0.3%	0.007
	Asymptomatic	6	4.1%	21	3.1%	7	1.0%	0.012
	Total^c^	10	6.8%	34	5.0%	9	1.3%	< 0.001
	No. tested	146		686		676		

^a^Two-tailed *P-*values for χ^2^ tests on 3 × 2 contingency tables testing the null hypothesis of similar proportions of positive results across rows.

^b^Pairwise comparisons of positivity rates across row with Fisher exact test (at 5% significance): index HHs > neighbor and control HHs; neighbor HHs = control HHs.

^c^Pairwise comparisons of positivity rates across row with Fisher exact test (at 5% significance): index and neighbor HHs > control HHs; index HHs = neighbor HHs.

Significantly different qPCR positivity rates were found across HH types, ranging from 6.8% to 15.0% in index HHs, 5.0% to 7.0% in neighbor HHs, and 0.3 to 5.6% in control HHs (Table 2). On days 0 and 30, positivity rates were significantly higher in index HHs (excluding the index cases) than in neighbor and control HHs (Fisher exact test, *P* < 0.001 in both comparisons) but they were similar when comparing neighbor to control HHs (Fisher exact test, *P* > 0.05 in both comparisons). Therefore, targeting neighbor HHs in search of new infections would not be more effective than randomly sampling community members on days 0 and 30. However, similar infection rates were found in index and neighbor HHs on day 60 (Fisher exact test, *P* = 0.231) and day 180 (Fisher exact test, *P* = 0.413). Furthermore, significantly higher infection rates were detected by qPCR in neighbor than control HHs in the last two RCD rounds (Fisher exact test, *P* < 0.001 in both comparisons).

### Household type, parasite density, and asymptomatic carriage

Microscopy diagnosed 1.9-fold and 1.2-fold more symptomatic than asymptomatic infections in index and neighbor HHs, respectively (Table 1), suggesting that many of these additional slide-positive subjects would be eventually detected by routine surveillance of febrile illnesses. Conversely, qPCR revealed 1.8-fold and 2.4-fold more asymptomatic than symptomatic infections in index HHs and neighbor HHs, respectively (Table 2). Moreover, 12.5-fold more asymptomatic than symptomatic infections were diagnosed by qPCR in control HHs (Table 2).

**Fig 4:**
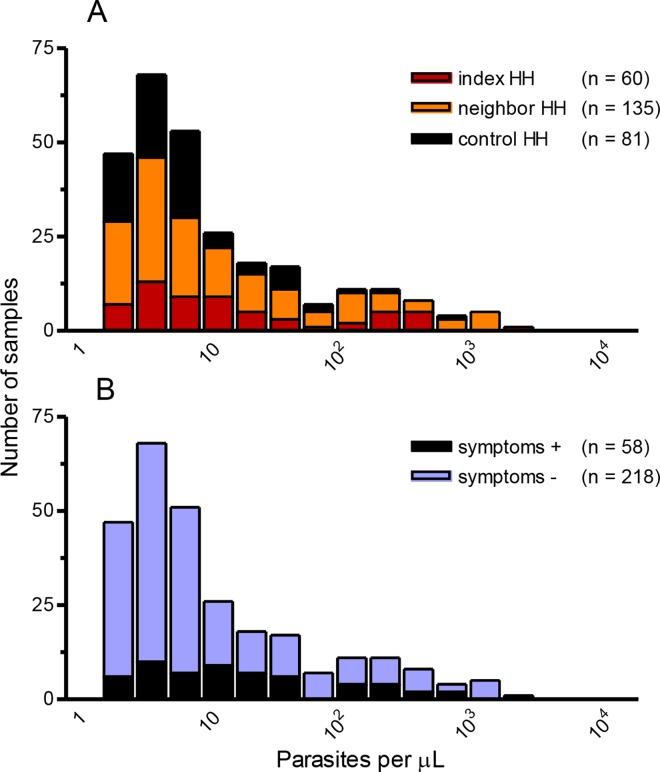
Frequency distribution of *Plasmodium vivax* blood-stage density classes (parasites per μL of blood in log scale). Parasitemias were estimated by quantitative real-time polymerase chain reaction (qPCR) and shown according to household (HH) type (panel A) and presence or absence of malaria-related clinical symptoms and signs (fever, headache, arthralgia, myalgia, and lower back pain) (panel B). The color code in panel A is the same as in Fig 1 to indicate index HHs, neighbor HHs, and control HHs.

We thus sought to estimate the relative contribution of index and neighbor HH members (who would be screened during RCD) and control HH members (who would be missed by RCD) to the total *P*. *vivax* biomass circulating in the study population. To this end, we used data from 276 qPCR-positive samples with available qPCR-based parasite-density measurements (Fig 4A). Overall, geometric mean parasitemias were twice as low in control HHs (5.6 parasites/μL [95% CI: 4.3–7.5]) as in index HHs (14.9 parasites/μL [95% CI: 8.9–24.7]) and neighbor HHs (11.8 parasites/μL [95% CI: 8.9–16.4]). Only 7.7% of the parasite biomass circulating in study subjects were contributed by control HH members (represented as black bar segments in Fig 4A), who had 27.4% of all qPCR-positive samples. The corresponding fractions harbored by index and neighbor HH members were 29.4% (20.7% of all qPCR-positive samples) and 62.8% (51.9% of all qPCR-positive samples) of the total parasite biomass, respectively.

Individuals with patent infections harbored 80.7% of the *P*. *vivax* biomass (geometric mean parasitemia, 34.6 parasites/μL [95% CI: 22.2–54.0]), while those that were missed by microscopy but detected by qPCR had much lower parasite densities (geometric mean parasitemia, 5.5 parasites/μL [95% CI: 4.6–6.7]). If conventional microscopy were the only diagnostic method available to identify infections and guide malaria treatment, a large proportion of the parasite biomass carried by index HH members (92.4%) and neighbor HH members (79.2%), but only 47.9% of that harbored by control HH members, would be detected and potentially removed by an effective antimalarial chemotherapy.

Parasite densities had a unimodal right-skewed frequency distribution in asymptomatic carriers (blue bar segments in Fig 4B); 65.6% of them were below 10 parasites/μL, with a geometric mean of 8.6 parasites/μL (95% CI: 6.8–10.8). Asymptomatic carriers contributed 75.4% of all qPCR-positive samples and 68.3% of the parasite biomass circulating in study subjects. Conversely, symptomatic subjects had a wide range of parasite densities (black bar segments in Fig 4B), with a nearly flat frequency distribution (geometric mean of 17.4 parasites/μL [95% CI: 10.6–28.4]); they contributed 26.1% of all qPCR-positive results and 31.7% of the parasite biomass. We next estimated the parasite biomass fraction that was harbored by control HH members who were asymptomatic, which would be missed by RCD-based strategies. We found that these subjects contributed 24.1% of all qPCR-positive results, but only 4.1% of the total *P*. *vivax* biomass measured in study subjects.

### Correlates of *P*. *vivax* infection diagnosed by microscopy and qPCR

We used mixed-effects logistic regression analysis to test whether the association between residence in the proximity to an index case and prevalence of *P*. *vivax* infection remained significant after controlling for potential individual- and HH-level confounders. The fully adjusted model revealed 56.7- and 18.5-times higher odds of slide-confirmed *P*. *vivax* infection among index and neighbor HH members, respectively, compared to control HH members (Table 3). Similarly, although with smaller magnitude than microscopy, the odds of qPCR-detected infection were significantly higher in index (OR = 3.40) and neighbor HH members (OR = 1.61), compared to control HH members (Table 3). Each additional year of residence in the area was associated with a 4% reduction (95% CI, 1–8%) in the risk of positivity by microscopy (*P* = 0.030) in the fully adjusted logistic model, thus regardless of the HH type. Interestingly, a history of slide-confirmed *P*. *vivax* infection within the past six months, in the fully adjusted model, was the only other variable significantly associated with increased risk of current infection detected by either microscopy (OR = 5.42; 95% CI: 3.36–8.75, *P* < 0.001) or qPCR (OR = 5.02; 95% CI: 2.77–9.09, *P* < 0.001). These findings suggest that selectively targeting individuals reporting recent *P*. *vivax* infections, regardless of their proximity to an index case, might be a practical way of finding current infections.

**Table 3:** Mixed-effects logistic regression analysis of the association between household (HH) type and risk of *Plasmodium vivax* infection diagnosed by microscopy or quantitative real-time polymerase chain reaction (qPCR) in Acrelândia, Brazil, 2013.

Diagnostic method	Regression model	Index HHs *vs*. Control HHs			Neighbor HHs *vs*. Control HHs		
		OR^a^	(95% CI)^b^	*P-*value	OR^a^	(95% CI)^b^	*P-*value
Microscopy^c^	Univariate	140.30	(30.8–639.7)	<0.001	30.00	(7.9–113.9)	<0.001
	Model 1^e^	141.90	(32.4–620.9)	<0.001	29.80	(8.1–110.6)	<0.001
	Model 2^f^	56.70	(13.9–231.1)	<0.001	18.50	(5.25–65.5)	<0.001
qPCR^d^	Univariate	5.67	(3.0–10.7)	<0.001	1.93	(1.3–3.0)	0.003
	Model 1^e^	5.95	(3.2–11.2)	<0.001	1.95	(1.3–3.0)	0.002
	Model 2^f^	3.40	(1.8–6.3)	<0.001	1.61	(1.1–6.3)	0.026

^a^OR = odds ratio.

^b^CI = confidence interval.

^c^Total of 5,866 observations across four rounds of reactive case detection.

^d^Total of 5,807 observations across four rounds of reactive case detection.

^e^Adjusted for age (continuous variable in years), gender, and HH size (<3, 3–5, >5 individuals). Likelihood-ratio test (univariate *vs*. Model 1), *LR* = 7.83 (*P* = 0.098) for microscopy, and *LR* = 4.39 (*P* = 0.356) for qPCR.

^f^Adjusted for all variables included on model 1 plus time of residence in the area (in years), overnight stay in the forest (yes vs. no), and history of slide-confirmed *P*. *vivax* infection within the past six months (yes vs. no). Likelihood-ratio test (Model 1 *vs*. Model 2), *LR* = 34.20 (*P* < 0.001) for microscopy, and *LR* = 50.61 (*P* < 0.001) for qPCR.

### *Plasmodium vivax* transmission networks around index cases

We used molecular genotyping data (S2 Database) to explore the epidemiological connections among infections diagnosed during the study. We found a high genetic diversity in local *P*. *vivax* populations, with 146 unique (predominant or only) genotypes found in 343 completely typed samples (267 from index and neighbor HHs and 76 from control HHs). Most (272 or 79.3%) subjects harbored mixtures of genotypes. Overall, six common genotypes accounted for 30.1% of all infections and were widely distributed across study localities (Table 4). The most common genotype (#81) was recovered from 54 (15.7%) study subjects living in all seven localities, being particularly prevalent (26.1% of all samples) in PTL. Moreover, we found no clear temporal clustering of the six most common genotypes; genotype #81 was recovered from samples collected year-round (S3 Fig).

**Table 4:** Distribution of the six most common *Plasmodium vivax* multilocus genotypes (that together accounted for 30.1% of all local infections) across seven study localities in Acrelândia, Brazil, 2013.

Genotype #^b^	Locality^a^ (number of samples completely typed)							
	PTL (*n* = 172)	PTD (*n* = 54)	ORI (*n* = 35)	SJB (*n* = 33)	SAP (*n* = 24)	RED (*n* = 21)	PEP *n* = 4)	All (n = 343)
61	7 (4.1%)^c^	1 (1.9%)	2 (5.7%)	1 (3.0%)	0 (0.0%)	0 (0.0%)	1 (25.0%)	12 (3.5%)
64	7 (4.1%)	1 (1.9%)	0 (0.0%)	2 (6.1%)	2 (8.3%)	0 (0.0%)	0 (0.0%)	12 (3.5%)
70	5 (2.9%)	0 (0.0%)	0 (0.0%)	0 (0.0%)	0 (0.0%)	2 (9.5%)	1 (25.0%)	8 (2.3%)
71	2 (1.2%)	3 (5.5%)	2 (5.7%)	1 (3.0%)	0 (0.0%)	0 (0.0%)	0 (0.0%)	8 (2.3%)
81	45 (26.1%)	2 (3.7%)	1 (2.9%)	3 (9.1%)	1 (4.2%)	2 (9.5%)	0 (0.0%)	54 (15.8%)
82	7 (4.1%)	0 (0.0%)	1 (2.9%)	0 (0.0%)	0 (0.0%)	1 (4.8%)	1 (25%)	10 (2.9%)
Others	99 (57.5%)	47 (87.0%)	29 (82.8%)	26 (78.8%)	21 (87.5%)	16 (76.2%)	1 (25.0%)	239 (69.7%)

^a^PTL = Gleba Porto Luiz, PTD = Reserva Porto Dias, ORI = Projeto Orion, SJB = Projeto São João do Balanceio (PTL), SAP = Projeto Santo Antônio Peixoto, RED = Projeto Redenção (RED), and PEP = Projeto Pedro Peixoto (PEP). The locations of these areas are shown in Fig 3.

^b^We considered only the predominant genotype found in each sample analyzed (see main text for further details).

^c^Percentages across columns were calculated for total number of samples analyzed within each locality

We next examined parasite genotypes within the 41 clusters surrounding index cases, each comprising one index HH and five neighbor HHs (see Fig 1). Between 1 and 21 (mean, 6.5) *P*. *vivax* infections were successfully genotyped per cluster during the four RCD rounds (n = 267 genotypes analyzed). Overall, 96 (35.9%) samples had genotypes that were either identical or similar to those found in other parasites within the same cluster, while 171 (64.0%) genotypes were unique (i.e., not shared by parasites within the same cluster). Therefore, nearly two thirds of all infections were genetically unrelated to each other within a putative malaria cluster. However, most (23 or 56.1%) clusters had at least one identical or similar genotype shared by two or more parasite samples.

**Fig 5:**
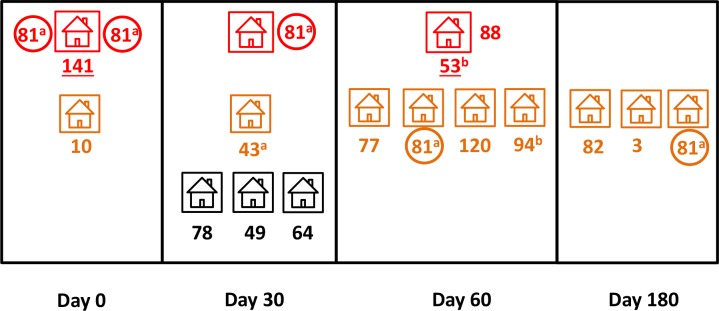
Shared and unique *Plasmodium vivax* multilocus genotypes within cluster #3 from Gleba Porto Luiz, Acrelândia, Brazil, during four rounds of reactive case detection (days 0, 30, 60, and 180). We used the same color code as in Fig 1 to indicate the index HH (red), neighbor HHs (orange), and control HHs (black). Predominant or only genotypes found in each sample are identified with numerals; those recovered from the index case are underlined. Predominant genotypes shared by ≥ 2 parasite samples within the cluster were circled; for example, two index HH members shared the same predominant genotype (#81) on day 0. Identical superscript letters indicate groups of samples harboring parasite lineages (e.g., parasites recovered on day 60 from the index case—whose major genotype is #53—and one neighbor HH member [whose major genotype is #94]) that were considered genetically similar when analyzing both major and minor alleles although not identical (see main text for definition).

Fig 5 shows several examples of shared and unique genotypes within cluster #3, located in PTL. Note that two index HH members shared the same genotype (#81) on day 0; one of them (who was not treated on day 0) still harbored this genotype 30 days later, consistent with a persisting blood-stage infection or an early homologous relapse, but acquired a different genotype (#88) detected on day 60. Haplotype #81 reappeared in two unrelated neighbor HH members on days 60 and 180, consistent with onward transmission originating from the index HH. Two instances of similar genotypes were also found in pairwise comparisons including mixed-clone infections; these cases are indicated with similar superscript letters in Fig 5. Finally, unique haplotypes (#3, 10, 77, 82, 88, 120, and 141) were found in seven samples, consistent with sporadic *P*. *vivax* infections that resulted in no further onward transmission within the cluster.

**Fig 6:**
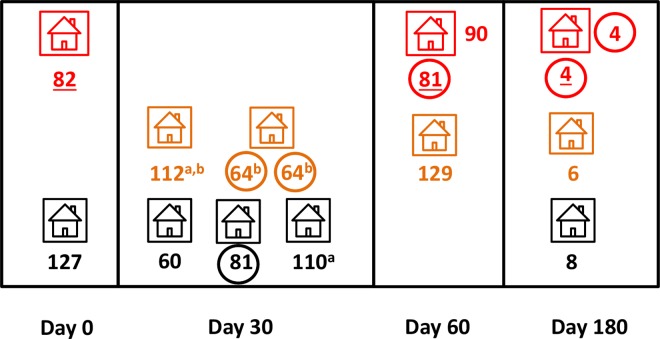
Shared and unique *Plasmodium vivax* multilocus genotypes within cluster #4 from Gleba Porto Luiz, Acrelândia, Brazil, during four rounds of reactive case detection (days 0, 30, 60, and 180). The index, neighbor, and control HHs are shown in red, orange, and black, respectively; predominant or only genotypes were identified with numerals and those recovered from the index case were underlined. Predominant genotypes shared by ≥ 2 parasite samples within the cluster were circled. Identical superscript letters indicate groups of samples harboring parasite lineages that were considered genetically similar although not identical (see main text for definition).

Examples of identical parasite strains within cluster #4 (also from PTL), suggesting a common source of infection, are shown in Fig 6: genotypes #64 (shared by two members of the same neighbor HH on day 30) and #4 (shared by the index case and another member of the index HH on day 180). The source of infection could not be determined in these cases. Interestingly, genotype #81 (which accounts for over one-fourth of all genotypes from PTL) was recovered from the index case (on day 60) and one control HH member (on day 30). The probability of finding genotype #81 in a pair of randomly chosen parasite samples from PTL, given by the squared frequency of this genotype in the local parasite population, is 6.8%. Similar genotypes were observed within the cluster as well as between members of index and control HHs (Fig 6).

We next examined all instances of recurrent infections within clusters. A total of 130 genotypes were recovered from 54 individuals who had parasite recurrences (range, 2 to 4) during the study. Genotypes recovered from most (41 or 75.9%) recurrent infections were different from those found in the primary infection, even when considering minor alleles in pairwise comparisons involving multiple-clone infections. These results are consistent with unrelated, sporadic infections or heterologous relapses. However, 11 (20.4%) subjects had one or more episodes of recurrent parasitemia with identical or similar genotypes and two (3.7%) had consecutive infections involving both identical and different genotypes (S1 Table). Overall, we found 17 instances of identical or similar genotype recurrence in these 13 subjects, 30–180 days after the primary infection, which may represent parasite recrudescences (≤ 30 days after the primary infection), homologous relapses after successful clearance of blood-stage parasites, or untreated, chronic blood-stage infections (see Fig 2 for definitions). Five subjects with genotype recurrences had subpatent primary infections that were left untreated, consistent with long-lasting infections being detected over the following RCD rounds (S1 Table).

**Fig 7:**
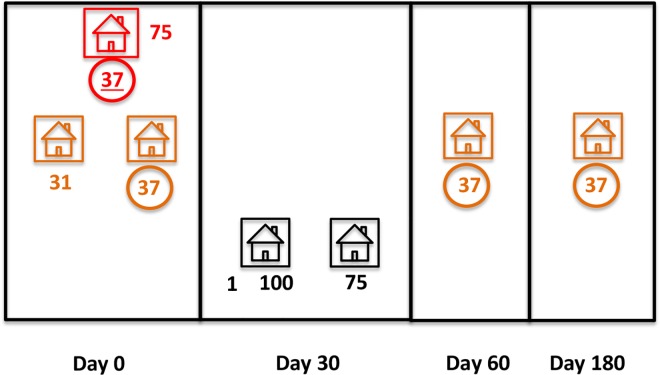
Shared and unique *Plasmodium vivax* multilocus genotypes within cluster #9 from Reserva Porto Dias, Acrelândia, Brazil, during four rounds of reactive case detection (days 0, 30, 60, and 180). The index, neighbor, and control HHs are shown in red, orange, and black, respectively; predominant or only genotypes were identified with numerals; the one recovered from the index case was underlined. Genotype #37 (circled) was recovered from four parasite samples, while genotype #75 was shared by an index HH member and a control HH member outside the cluster.

Fig 7 shows an example of recurrent infection with the same genotype within cluster #9, from PTD. Genotype #37 was first recovered from the index case and one neighbor HH member on day 0 (consistent with a common source of infection; Fig 2). This same neighbor HH member, whose primary infection treated, had recurrent subpatent parasitemia with genotype #37 on days 60 and 180, consistent with homologous parasite relapses.

Only six control HH members presented qPCR-detected parasite recurrences during the study. In all cases, different genotypes were found in the primary and subsequent infection. Although all infections were subpatent and left untreated, we found no example of chronic infection with the same strain reappearing in consecutive visits to control HH members.

## Discussion

Malaria burden in Brazil has reached its lowest levels in more than three decades. This success led the Ministry of Health to launch, in November 2015, a Plan for Elimination of Malaria in Brazil, in alignment with the new Sustainable Development Agenda of the United Nations aimed to reduce the global number of malaria cases by 90% until 2030, and to eventually eliminate malaria in 35 countries [7]. To this end, operationally feasible strategies that can address the vast parasite reservoir found in residual malaria pockets are urgently needed. In this study, we evaluated a RCD strategy tailored for rural Amazonian communities with low *P*. *vivax* endemicity and spatial clustering of malaria episodes.

Our results provided further evidence that *P*. *vivax* infections are strongly clustered at the HH level in rural Amazonia [12,13]. In fact, over 53% of all index HHs are located in a single locality (PTL) in the study site (Fig 3). Such a clustering indicates that RCD programs targeting index HHs may be effective in detecting a large proportion of parasite carriers, many of them asymptomatic, in high-risk HHs. Also, index HH members persisted at increased risk of infection over up to 180 days after the index case diagnosis, suggesting that several rounds of RCD may be required to detect a large proportion of new infections within these clusters. These data suggest that malaria transmission hotspots in the area are stable over time, indicating that their identification may be used to target high-priority HHs for control and elimination. The finding that a history of slide-confirmed malaria within the past six months is a strong predictor of *P*. *vivax* positivity in all RCD rounds suggests a simple way of identifying high-risk subjects that can be further explored in the design of narrowly focused and intensive active surveillance strategies. In addition, although qPCR detected nearly twice more infections than conventional microscopy in index HHs, subpatent infections contributed only 7.6% of the total *P*. *vivax* biomass circulating in index HH members. Combined, these findings suggest that a highly sensitive molecular method is not an essential component of RCD programs targeting index HH members. Interestingly, the number of slide-confirmed *P*. *vivax* infections diagnosed in Acrelândia declined substantially after our study was completed; 117 and 54 *P*. *vivax* infections (99 and 40 of them classified as autochthonous) were recorded in this municipality in 2014 and 2015, respectively (http://portalsaude.saude.gov.br/index.php/o-ministerio/principal/leia-mais-o-ministerio/662-secretaria-svs/vigilancia-de-a-a-z/malaria/11346-situacao-epidemiologica-dados).

Large-scale RCD programs have been either limited to index HHs or extended to other HHs in the vicinity of the index HHs [28,29]. It has been hypothesized that limiting RCD to index HHs is cost-effective in areas where routine malaria surveillance already detects a large proportion of positive HHs; however, actively screening the surrounding HHs for new infections may be required in areas with a low coverage of passive surveillance [30]. Comparisons across studies are limited by the use of different criteria to define and recruit “neighbors” (e.g., predefined radius around index cases vs. predefined number of nearest HHs; all vs. febrile neighbors only) and by the varying biological features (e.g., flight range) of local vectors [27,28].

Here we show that malaria positivity rates among neighbor HH members on day 0, when the index case diagnosis triggered the initial investigation, were not significantly different from those found in randomly selected inhabitants in the same communities. Therefore, targeting neighbor HH members at the time of index case diagnosis did not appear to be more cost-effective than screening randomly chosen individuals for identifying new infections in our rural Amazonian setting. Nevertheless, much higher microscopy positivity rates were found in neighbor HHs, compared to control HHs, over the next months, with a similar trend observed for qPCR-based diagnosis in the last two RCD rounds. Furthermore, nearly two-thirds of the *P*. *vivax* biomass harbored by study participants was found in neighbor HH members, highlighting their potential contribution to ongoing malaria transmission in the community. One possible explanation for the increased prevalence of infections in neighbor HH members diagnosed on days 60 and 180 is that at least some of them may be secondary to those diagnosed in index HH members on days 0 and 30. We thus conclude that neighbor HHs must be screened in RCD programs in endemic settings similar to our study site. Defining the appropriate timing for microscopy- or qPCR-based screening is crucial for finding these additional cases in a cost-effective way.

A major limitation of RCD-based strategies is that asymptomatic parasite carriers will be missed if no single symptomatic infection is found in their vicinity. A recent survey in coastal Kenya, for example, has revealed different features of clusters of asymptomatic vs. symptomatic infections, such as the greater temporal stability of the former over > 10 years; these clusters with purely asymptomatic or some symptomatic infections did not necessarily overlap spatially in all localities [31]. qPCR-detected asymptomatic infections in our control HHs, which would be entirely missed by RCD, contributed a small fraction (4.1%) of the total parasite biomass found in study participants. Their infectiousness to local vectors remains unknown; one study described an infection rate of 1.2% after feeding *An*. *darlingi* with blood from asymptomatic *P*. *vivax* carriers vs. 22% for the mosquitoes fed with blood from symptomatic carriers [32]. Under the assumption that numbers of circulating *P*. *vivax* asexual blood-stages and mature gametocytes are positively correlated to each other [33], asymptomatic carriers of low-level parasitemias are expected to have relatively few circulating mature gametocytes and thus contribute relatively little to ongoing malaria transmission at a particular time point. However, they may represent a large number of infective man-days if they remain parasitemic for weeks or months and gametocytemias reach a critical (yet undetermined) density threshold for successful mosquito infection [34,35]. Reassuringly, in our study population we found very few asymptomatic infections persisting over consecutive visits to control HHs, none of them with the same genotype, suggesting that most episodes of asymptomatic carriage were short-lived. Whether periodic mass blood screenings with highly sensitive diagnostic methods would be a cost-effective way of addressing this occult parasite reservoir in malaria elimination programs remains to be determined. Alternatively, molecular diagnostic methods in elimination programs may be carefully targeted at subjects who are found to be at high risk of persistent asymptomatic and subpatent parasitemia (e.g., young adults, forest workers etc.) in sequential surveys of a given population.

Malaria clusters in our study localities were not synonymous with isolated, common-source outbreaks involving one or a few closely related parasite genotypes, as in recently described *P*. *falciparum* outbreaks in Peru [36] and Panama [37]. To the contrary, we characterized a highly diverse *P*. *vivax* population in our study site as well as in endemic settings nearby [38–40], with a wide variety of strains co-circulating not only in the proximity of index cases but also in the surrounding HHs outside the clusters. Accordingly, nearly two thirds of the genotypes circulating within each cluster were unique, consistent with a continuous introduction of new parasite strains and little onward transmission of these strains to neighboring individuals over the following months. These results suggest that a substantial proportion of infections are acquired outside the operationally defined malaria clusters; alternatively, human movement may continuously spread parasite genotypes (wherever infections were acquired) beyond the limits of the clusters. In other words, clusters would not represent isolated islands but archipelagos with considerable parasite migration between islands. Moreover, different strains were characterized in three-fourths of parasite recurrences observed in 54 study subjects, suggesting that re-infections or heterologous relapses, rather than recrudescences, homologous relapses, or chronic untreated blood-stage infections, are responsible for most repeated episodes of parasite carriage diagnosed within malaria clusters during the four RCD rounds.

Results shown here may help decision-makers to design malaria elimination strategies in low-endemicity settings where *P*. *vivax* largely predominates, such as most Latin American countries [41]. We conclude that RCD-based strategies targeting index HH members and their neighbors may be suggested as a major component of malaria elimination efforts in Brazil. Whether the subpatent and asymptomatic carriers who remain outside the identified clusters of symptomatic infections, beyond the reach of RCD programs, may represent a significant parasite reservoir remains to be determined. These silent infections could only be identified by mass blood surveys using highly sensitive diagnostic techniques, whose feasibility and cost-effectivity are currently unknown. Finally, genotyping data have revealed rather complex networks of residual malaria transmission in rural Amazonian communities, consistent with multiple sources of infection, further complicating current elimination efforts.

## Supporting Information

S1 Checklist STROBE (STrengthening the Reporting of OBservational studies in Epidemiology) Checklist. As required for all observational studies published by *PLoS Neglected Tropical Diseases*, this paper includes the STROBE checklist to document its compliance with STROBE guidelines.
(PDF)

S1 Fig Map showing the location of the study site, the municipality of Acrelândia, in the State of Acre, southwestern part of the Amazon Basin of Brazil. The map also indicates the location of the nearest towns, Acrelândia, Plácido de Castro, Senador Guiomard, and Rio Branco (capital of Acre), and the BR 364 interstate highway, which connects the States of Acre, Rondônia, and southern Amazonas to the rest of the country.
(PDF)

S2 Fig Home visits to study participants in Acrelândia, Brazil, 2013. Photographs by Alessandra Fratus.
(PDF)

S3 Fig Temporal distribution of the six most common *Plasmodium vivax* multilocus genotypes (that together accounted for 30.1% of all local infections) in study localities in Acrelândia, Brazil, 2013. (PDF)

S1 Table Multilocus genotypes in consecutive *Plasmodium vivax* infections diagnosed in 54 study subject during reactive case detection rounds (days 0, 30, 60, and 180) in Acrelândia, Brazil, 2013. (PDF)

S1 Database Excel file with all variables used in the logistic regression models. (XLSX)

S2 Database Excel file with genotyping data from 343 completely analyzed *P*. *vivax* infections. (XLSX)
