# Supplementary material for: Reactive Case Detection for Plasmodium vivax Malaria Elimination in Rural Amazonia
Source: PLoS Negl Trop Dis. 2016 Dec 12;10(12):e0005221. doi: 10.1371/journal.pntd.0005221 (PMC5179126; doi:10.1371/journal.pntd.0005221)
Supplement: S1 Fig — The map also indicates the location of the nearest towns, Acrelândia, Plácido de Castro, Senador Guiomard, and Rio Branco (capital of Acre), and the BR 364 interstate highway, which connects the States of Acre, Rondônia, and southern Amazonas to the rest of the country. (PDF) [file pntd.0005221.s002.pdf]

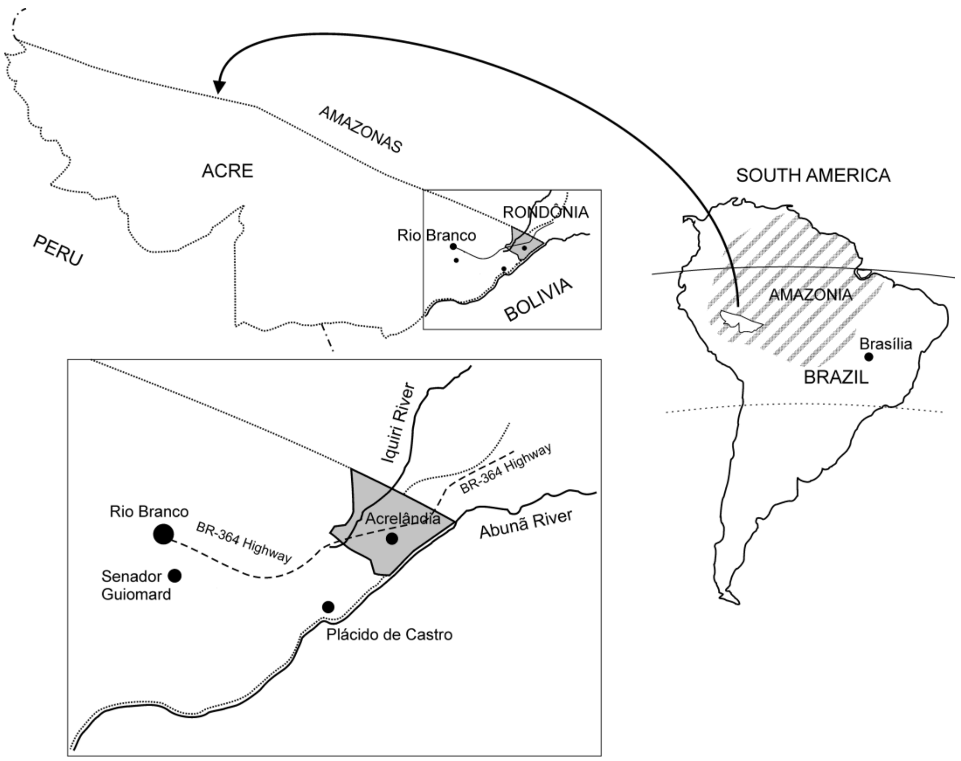

Figure S1. Map showing the location of the study site, the municipality of Acrelândia, in the State of Acre, southwestern part of the Amazon Basin of Brazil. The map also indicates the location of the nearest towns, Acrelândia, Plácido de Castro, Senador Guiomard, and Rio Branco (capital of Acre), and the BR 364 interstate highway, which connects the States of Acre, Rondônia, and southern Amazonas to the rest of the country.
