# Supplementary material for: Reactive Case Detection for Plasmodium vivax Malaria Elimination in Rural Amazonia
Source: PLoS Negl Trop Dis. 2016 Dec 12;10(12):e0005221. doi: 10.1371/journal.pntd.0005221 (PMC5179126; doi:10.1371/journal.pntd.0005221)
Supplement: S2 Fig — Photographs by Alessandra Fratus. (PDF) [file pntd.0005221.s003.pdf]

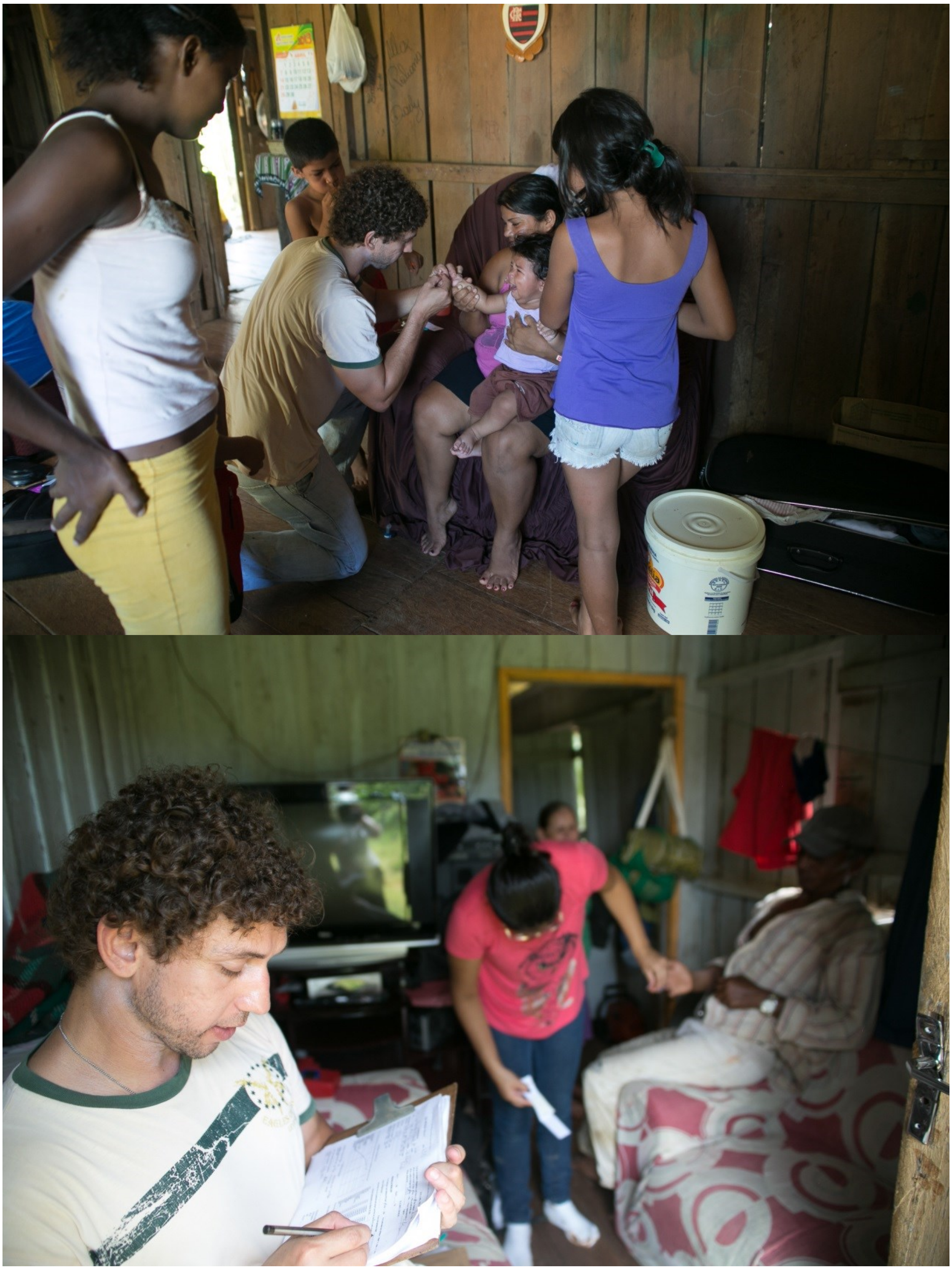

Figure S2. Home visits to study participants in Acrelândia, Brazil, 2013. Photographs by Alessandra Fratus. The photographer identified herself and the purpose of the photograph to the people being photographed and the individuals agreed to have their photograph taken and potentially published.
