# Supplementary material for: Reactive Case Detection for Plasmodium vivax Malaria Elimination in Rural Amazonia
Source: PLoS Negl Trop Dis. 2016 Dec 12;10(12):e0005221. doi: 10.1371/journal.pntd.0005221 (PMC5179126; doi:10.1371/journal.pntd.0005221)
Supplement: S3 Fig — (PDF) [file pntd.0005221.s004.pdf]

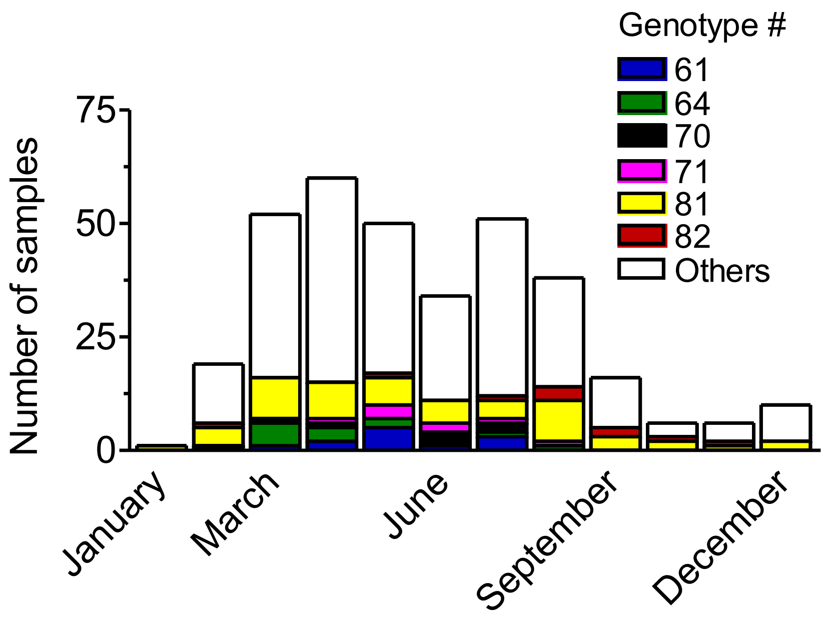

Figure S3. Temporal distribution of the six most common *Plasmodium vivax* multilocus genotypes (that together accounted for 30.1% of all local infections) in study localities in Acrelândia, Brazil, 2013.
