# Supplementary material for: Reactive Case Detection for Plasmodium vivax Malaria Elimination in Rural Amazonia
Source: PLoS Negl Trop Dis. 2016 Dec 12;10(12):e0005221. doi: 10.1371/journal.pntd.0005221 (PMC5179126; doi:10.1371/journal.pntd.0005221)
Supplement: S1 Table — (PDF) [file pntd.0005221.s005.pdf]

Table S1. Multilocus genotypes in consecutive *Plasmodium vivax* infections diagnosed in 54 study subjects during reactive case detection (RCD) rounds (days [d] 0, 30, 60, and 180) in Acrelândia, Brazil, 2013.

				msp1F3		*pv3.27*		*MS16*		
**Cluster**	**Subject**	**RCD round**	**Month**	**A1**	**A2**	**A1**	**A2**	**A1**	**A2**	**Comparison within group (most plausible interpretation)**
1	1	d0*	January	205		216		255		Different genotypes
		d60*	April	190		204	208	276	189	
	14	d60*	April	190		220		276	156	Different genotypes
		d180	August	190		208		276		
2	30	d0*	February	208		300		300	174	Different genotypes
		d180	August	205		208	300	300		
3	34	d0*	February	235	190	208		276		Different genotypes
		d60	April	205	151	204	152	126		
	36	d0	February	205		216		255		Identical genotypes on d0 and d30
		d30	March	205		216		255		(persisting untreated infection or
		d60*	April	205		220		255		early relapse); different on d60
	47	d60	April	205	250	216		171	255	Different genotypes
		d180	August	205	151	216		258		
4	52	d0*	February	205		216		258		Different genotypes
		d60*	April	205		216	228	255		
		d180*	August	106	205	208		255		
	53	d60	April	205	271	228	216	198	252	Different genotypes
		d180	August	106	217	208		255		
	435	d30	March	208	205	208	216	300	174	Different genotypes
		d60	April	202		208		189	276	

5	42	d0*	February	205	202	208	220	255		Different genotypes
		d180*	August	205		216		255		
	92	d30*	March	205		216		255		Identical genotypes on d30, 60, and
		d60*	April	250	205	216		255		d180 (recrudescences or relapses)
		d180*	August	205		216	228	255		
	105	d30*	March	205		216		255		Identical genotypes
		d60*	April	205	202	216	224	255	165	(recrudescence or early relapse)
6	70	d30*	March	205		216	204	255		Identical genotypes on d30, 60, and
		d60*	April	205		208	216	255	183	d180 (recrudescences or relapses)
		d180*	August	205		216		255		
	71	d0*	February	226	151	208	216	297	300	Different genotypes
		d30*	March	205		216		234		
		d60*	April	226		236		204		
	72	d60	April	205		236	228	258		Different genotypes
		d180*	August	202		224	216	258		
	341	d30	March	205		216		174	156	Different genotypes
		d60*	April	205		216	224	156	204	
	343	d60*	April	205	226	220		255		Different genotypes
		d180*	August	205	226	212		255		
8	118	d0*	February	202	247	216		174		Different genotypes
		d30*	March	202	226	252		276		
	214	d30*	March	205		208		174		Different genotypes
		d60	May	205		208		300		
9	335	d0*	February	202		208		276		Identical genotypes
		d60*	May	202		208		276	189	(relapses)
		d180	August	202		208	216	276		

10	334	d60*	May	205	226	256	216	255		Different genotypes
		d180*	September	205	250	208		258		
12	280	d0*	March	205	226	216		255		Different genotypes
		d30*	April	205		252		228		
	281	d0*	March	205	229	216		255		Identical genotypes (late relapse)
		d180	September	205		228	216	255	195	
14	453	d30	April	205	226	216	204	255		Identical genotypes on d30 and d180
		d60	May	205		220		255		(persisting untreated infection or late
		d180	September	205		204		255		relapse), but different on d60
15	323	d60*	June	202	199	208		276		Different genotypes
		180*	September	202		208	216	255		
	328	d30*	April	202	205	204		273		Different genotypes
		d60*	May	208	205	208	216	276	126	
		d180	September	217		248		303	207	
	330	d30*	April	205		280	204	174		Different genotypes
		d60*	May	202		208		189		
	331	d30*	April	205		220		255		Different genotypes
		d60*	May	202	247	220		255		
		d180*	September	220		252		207	303	
18	88	d0*	April	205	211	208	216	126	111	Different genotypes
		d60*	June	211	208	216		156		
19	547	d0*	April	205		216		255		Different genotypes
		d30*	May	205		320	232	255		
20	565	d0*	April	205		216		255		Different genotypes
		d30*	May	109	130	208	216	129		
		d60*	June	211		204	252	123		

		d180*	October	205	151	200		300		
21	625	d0*	April	202		208		303		Different genotypes
		d30*	May	217		276		174		
23	737	d0	May	205		216		255		Identical genotypes (persisting
		d180	November	205		216	208	255		untreated infection or late relapse)
	748	d0	May	205	202	216		255		Different genotypes
		d180	November	205	151	208	212	204		
24	800	d0*	May	205	226	216		255		Different genotypes
		d180	November	205		216		258		
26	841	d0	May	202		208		243		Different genotypes
		d30	June	202		212		276		
27	857	d0	May	205		216		255	183	Identical genotypes (persisting
		d60*	July	205		216	228	255	198	untreated infection or relapse)
	1393	d30	June	106	124	208		129		Different genotypes
		d180*	November	205		228	216	255		
	1398	d30	June	205	151	204	252	126		Different genotypes
		d60	July	205		216		255		
28	93	d0*	May	205		220		255	183	Different genotypes
		d60	July	205		216		255		
30	868	d0*	May	205		216		255		Identical genotypes (relapse)
		d60	August	205		216	208	255	123	
	869	d30*	July	205	202	204	216	255		Identical genotypes (recrudescence
		d60*	August	205		216		255		or late relapse)
		d180*	December	205		216		255		
	921	d0	May	217		216		174		Different genotypes
		d180	December	106		208	192	204		

	1539	d30	July	205		252		276		Different genotypes
		d60	August	205	190	204		123		
31	878	d0*	May	217		228		264	156	Different genotypes
		d30*	July	106	124	208		129		
	881	d30*	July	226	205	204	226	303		Different genotypes
		d60*	August	202	256	208		207	303	
	884	d60*	August	202		208		207	201	Different genotypes
		d180	December	202		208		303		
34	2004	d0*	June	205		216	204	255	216	Different genotypes
		d60*	August	199		228		255		
35	1025	d0	June	202		208		303		Different genotypes
		d30	July	205		216		276		
36	1098	d0*	June	205		216	204	255		Different genotypes
		d30	July	190	211	208		276		
38	1261	d0*	July	235	190	208	188	276		Different genotypes
		d30*	August	205	202	208	216	276		
39	1309	d0*	July	208	229	228	208	177	183	Different genotypes
		d30	August	205		208		174		
40	1350	d0*	July	205		216		255	183	Identical genotypes (relapse)
		d60	September	205		216		255	213	
41	1561	d0*	July	202		208	204	273	228	Identical genotypes (recrudescence
		d30*	August	202	226	204	226	273	228	or early relapse)

Notes: A1 and A2 denote, respectively, the sizes (in base pairs) of the major (most abundant) and minor allele (if any) at a given locus. Identical alleles (either major or minor) in consecutive samples from the same patient are highlighted. Asterisks (RCD round column) indicate infections treated with a standard regimen of chloroquine and primaquine. By defining a pair of genotypes as “different” we mean no allele sharing was found at any locus, considering both A1 and A2.
